# An electrochemiluminescence resonance energy transfer biosensor for the detection of circulating tumor DNA from blood plasma

**DOI:** 10.1016/j.isci.2021.103019

**Published:** 2021-08-21

**Authors:** Xidong Yang, Meiyan Liao, Hanfei Zhang, JinBo Gong, Fan Yang, Mengying Xu, Pier-Luc Tremblay, Tian Zhang

**Affiliations:** 1 School of Chemistry, Chemical Engineering, and Life Science, Wuhan University of Technology, Wuhan 430070, PR China; 2 Shaoxing Institute for Advanced Research, Wuhan University of Technology, Shaoxing 312300, PR China; 3 Department of Radiology, Zhongnan Hospital of Wuhan University, Wuhan 430071, China; 4 State Key Laboratory of Silicate Materials for Architectures, Wuhan University of Technology, Wuhan 430070, PR China; 5 School of Materials Science and Engineering, Wuhan University of Technology, Wuhan 430070, PR China

**Keywords:** Sensor, Optical imaging, Biomolecules

## Abstract

A liquid biopsy is a noninvasive approach for detecting double-stranded circulating tumor DNA (ctDNA) of 90–320 nucleotides in blood plasma from patients with cancer. Most techniques employed for ctDNA detection are time consuming and require expensive DNA purification kits. Electrochemiluminescence resonance energy transfer (ECL-RET) biosensors exhibit high sensitivity, a wide response range, and are promising for straightforward sensing applications. Until now, ECL-RET biosensors have been designed for sensing short single-stranded oligonucleotides of less than 45 nucleotides. In this work, an ECL-RET biosensor comprising graphitic carbon nitride quantum dots was assessed for the amplification-free detection in the blood plasma of DNA molecules coding for the EGFR L858R mutation, which is associated with non-small-cell lung cancer. Following a low-cost pre-treatment, the highly specific ECL-RET biosensor quantified double-stranded EGFR L858R DNA of 159 nucleotides diluted into the blood within a linear range of 0.01 fM to 1 pM, demonstrating its potential for noninvasive biopsies.

## Highlights

• An ECL-RET biosensor with g-CNQDs was developed for liquid biopsies of ctDNA
• The biosensor detected DNA molecules coding for the lung cancer EGFR L858R mutation
• EGFR L858R DNA molecules of 18 and 159 nucleotides activated the biosensor
• The biosensor detected ctDNA-like EGFR L858R molecules diluted in blood plasma

## Graphical abstract

**Figure:**
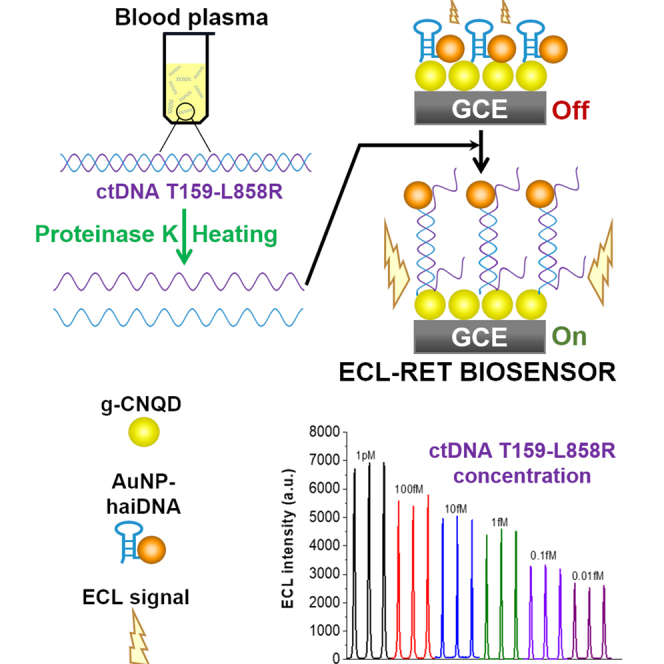

## Introduction

The blood plasma of patients with cancer often harbors double-stranded (ds) circulating tumor DNA (ctDNA) released from necrotic or apoptotic tumor cells (van Ginkel et al., 2017; Jahr et al., 2001). The nucleotide sequence of ctDNA comprises mutations specific to different types of cancer (Iwahashi et al., 2019; Han et al., 2019; Said et al., 2020; Reece et al., 2019). This attribute can be exploited for the non-invasive detection of early- to late-stage cancers and to monitor treatment efficiency via liquid biopsy of blood samples (Del Re et al., 2019; Chen et al., 2020b; Kelley and Pantel, 2020). Standard techniques for detection and quantification of ctDNA by liquid biopsy include quantitative PCR (qPCR) and droplet digital PCR (ddPCR) with probes recognizing specific cancer-related mutations, as well as next-generation sequencing (NGS) (Chen and Zhao, 2019; Karachaliou et al., 2017; Hrebien et al., 2019; Busser et al., 2017). To simplify these bioassays and reduce cost and processing time, other techniques are under development such as surface plasmon resonance imaging and electrochemical sensors, which exhibit various degrees of sensitivity to specific ctDNA molecules (Gorgannezhad et al., 2018; Das et al., 2016; Noh et al., 2015; Chu et al., 2016; Yuanfeng et al., 2020; Huang et al., 2020; Soda et al., 2019; Li et al., 2020; D'Agata et al., 2020; Bellassai et al., 2021; Luo et al., 2021). In the case of electrochemical systems, many of them rely on signal amplification often via enzymatic processes, which increases the complexity of sample handling as well as the overall cost of the assay (Das et al., 2015; Hu et al., 2021; Wang et al., 2018; Li et al., 2017).

In recent years, liquid biopsy has been developed for the diagnosis of non-small-cell lung cancer (NSCLC) (Krug et al., 2018; Oxnard et al., 2014). Eighty-five percent of lung cancer, which is the main cause of cancer-related death in the world, is NSCLCs (Reck et al., 2014; Malvezzi et al., 2015). Mutations in the epidermal growth factor receptor (EGFR) are often involved in the development of NSCLC-related tumors (Bethune et al., 2010). Amino acid replacement L858R in exon 21 and deletions in exon 19 account for more than 80% of EGFR mutations (Sakurada et al., 2006). These modifications in EGFR result in an increase of the tyrosine kinase activity and facilitate cell proliferation as well as metastasis (Lynch et al., 2004). The detection of EGFR mutations via tumor tissue biopsy is a routine procedure for the diagnosis of cancer (Alegre et al., 2016). However, this approach is invasive and not risk free for patients. Thus, research efforts have been deployed to develop molecular techniques for the detection and quantification of EGFR-related ctDNA in blood samples (Heitzer et al., 2015).

**Scheme 1:**
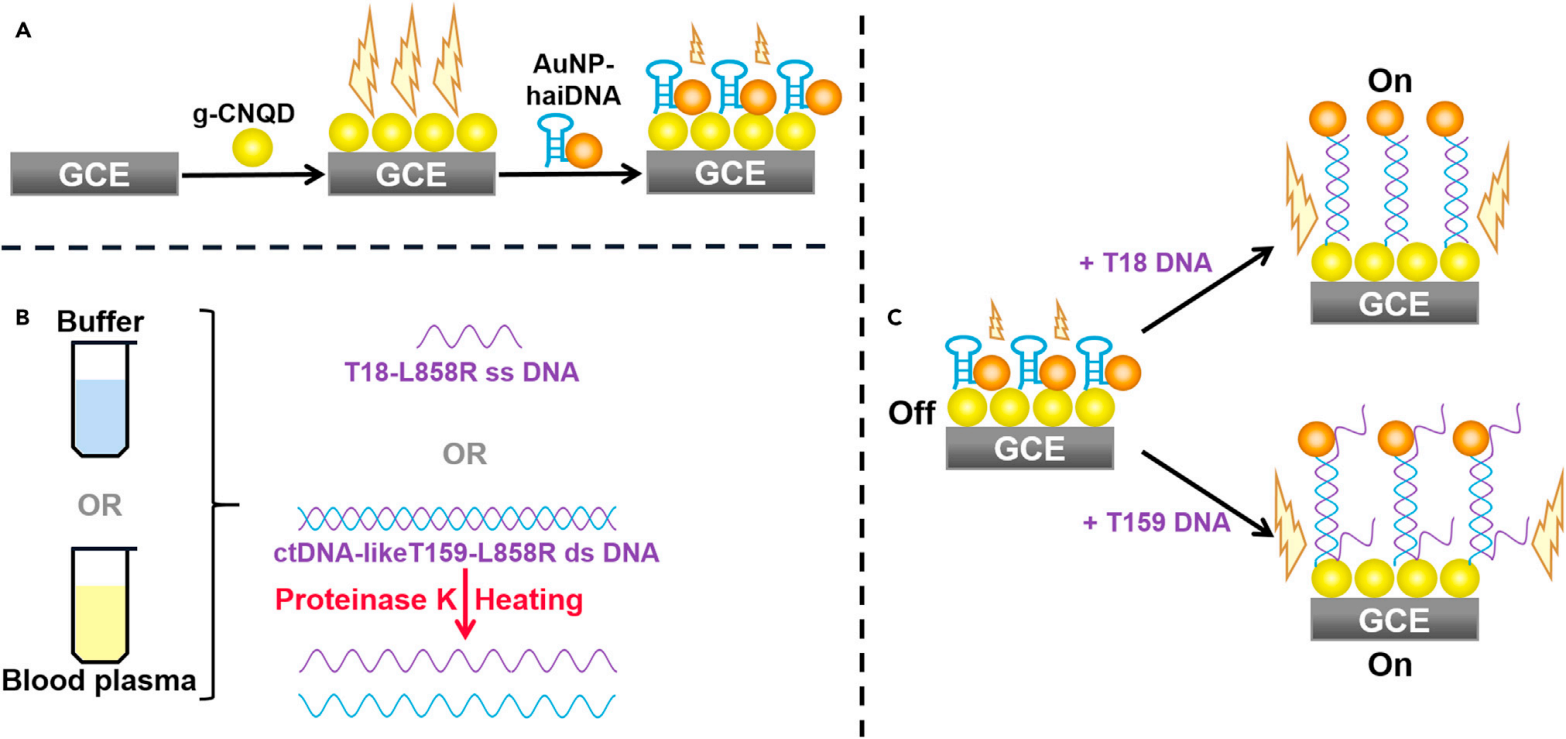
Principle of the ECL-RET biosensing assay for the detection of cancer-related EGFR L858R target DNA molecules (A) Fabrication of the ECL-RET biosensor with g-CNQDs and AuNP-haiDNA probes. The strong ECL signal emitted by g-CNQDs on the GCE is quenched by proximal AuNPs attached to the haiDNA probes. (B and C) Sample preparation. T18-L858R ss oligonucleotides are diluted into potassium phosphate buffer. CtDNA-like T159-L858R ds DNA molecules are diluted in potassium phosphate buffer or in blood, which is subsequently centrifuged to separate plasma. For T159-L858R ds DNA molecules, plasma samples are treated with proteinase K and then heated prior to (C) detection with the ECL-RET biosensor. In the presence of target DNA molecules, haiDNA probes anneal with them increasing the distance between AuNPs and g-CNQDs and the intensity of the ECL signal.

Electrochemiluminescence resonance energy transfer (ECL-RET) biosensor is a promising technology with high sensitivity and a large concentration response range for the detection and quantification of different analytes including nucleic acids, antigens, metabolites, and whole cells (Chen et al., 2014; Chen et al., 2019; Chen et al., 2020a; Tian et al., 2012; Wu et al., 2011; Zhang et al., 2008; Martínez-Periñán et al., 2020). In ECL-RET biosensors designed for DNA detection, semiconductor quantum dots (QDs) such as eco-friendly SiQDs or graphitic-carbon nitride QDs (g-CNQDs) coated on a glassy carbon electrode (GCE) generate an electrochemiluminescence (ECL) signal (Scheme 1) (Liu et al., 2019; Dong et al., 2017). Hairpin DNA probes modified with Au nanoparticles (AuNP-haiDNA) are attached to the surface of the coated GCE. In the absence of target DNA, Au nanoparticles (AuNPs) are near the coated GCE surface and quench the ECL signal. When target DNA molecules are present in the analyzed solution, they anneal to the AuNP-haiDNA probes, which augments the distance between AuNPs and semiconductor QDs resulting in increased ECL signal. Until now, ECL-RET biosensors for nucleic acids have been developed and focused mainly on the detection of single-stranded (ss) DNA molecules shorter than 45 nucleotides diluted in phosphate-buffered saline or other well-defined buffer solutions (Zhang et al., 2008; Zhou et al., 2012; Liu et al., 2019; Dong et al., 2017).

In this proof-of-concept study, an amplification-free ECL-RET biosensor-based assay was developed for the detection and quantification in the blood plasma of ds DNA molecules harboring the single-nucleotide polymorphism (SNP) responsible for the EGFR L858R mutation. When compared with commercial PCR- or NGS-based techniques for liquid biopsy, ECL-RET biosensors could exhibit several advantages such as requiring no expensive DNA extraction and purification kit for sample pre-treatment as well as being easier to operate (Tables S1 and S2). The biosensing assay designed here with g-CNQDs was employed to detect short 18-nucleotide ss DNA and longer 159-nucleotide ds DNA in both potassium phosphate buffer and blood.

## Results and discussion

### g-CNQD-based ECL-RET biosensor for cancer-related DNA

**Figure 1:**
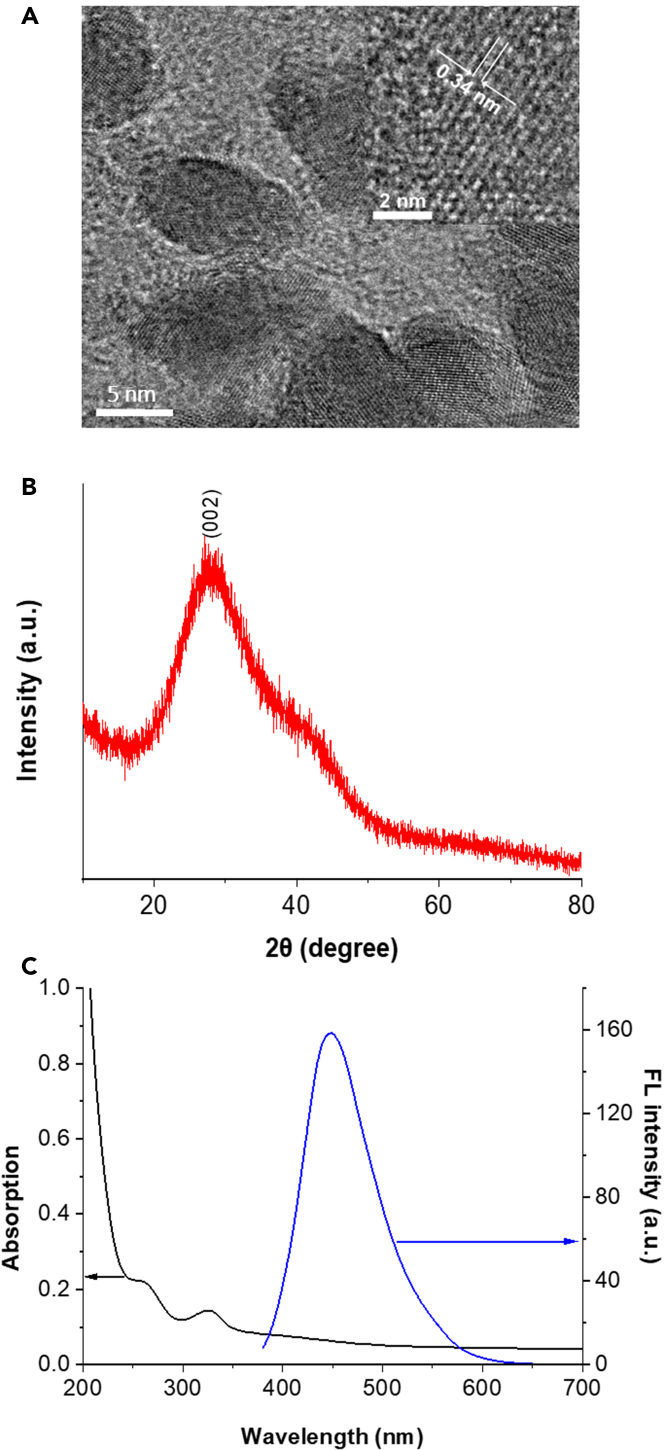
Characterization of g-CNQDs (A–C) TEM images, (B) XRD, (C) UV-Vis absorption, and steady-state fluorescence spectra of g-CNQDs.

The components and working principles of the ECL-RET biosensor system developed here for sensing DNA molecules carrying cancer-related mutations are described in Scheme 1. The first step of the assembling of the g-CNQD-based ECL-RET biosensor, which is the deposition of g-CNQDS on the GCE, has been reported previously by Liu et al. and is only briefly described here (Liu et al., 2019). The quality of the synthesized g-CNQDs was evaluated by transmission electron microscopy (TEM), Fourier transform infrared (FTIR) spectroscopy, X-ray diffraction (XRD) analysis, UV-Vis absorption spectrophotometry, and fluorescence spectroscopy (Figures 1; S1). The TEM image indicates that the synthesized g-CNQDs were oval with suitable uniformity and a diameter ranging from 3.8 nm to 11.3 nm with an average particle size of 6.5 nm (n = 30) (Figures 1A; S2). The inset in Figure 1A shows an interlayer spacing of 0.34 nm characteristic of the (002) plane of hexagonal graphitic carbon nitride. The pattern of peaks on the FTIR spectrum is distinctive of g-CNQDs (Figure S1) (Zhou et al., 2013; Liu et al., 2011). The XRD spectrum of g-CNQDs exhibits a strong diffraction peak at 27.6° (002) consistent with previous reports as well as with the interlayer spacing identified by TEM (Figure 1B) (Zhang et al., 2019; Abdolmohammad-Zadeh and Rahimpour, 2016). Furthermore, the UV-Vis spectrum shows an absorption peak characteristic of g-CNQDs at 335 nm (Figure 1D) (Zhou et al., 2013). The outstanding optical characteristic g-CNQDs were confirmed by the steady-state fluorescence spectrum (Figure 1C). Upon excitation of g-CNQDs at 365 nm, an intense symmetrical peak at 450 nm was generated.

**Figure 2:**
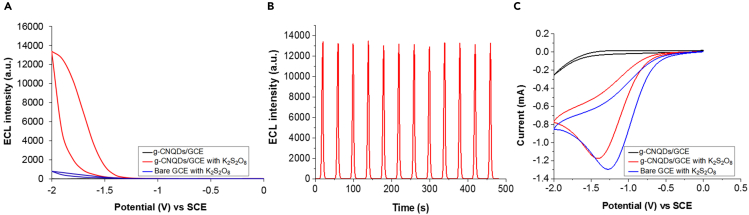
The ECL behavior of g-CNQDs on GCE with K_2_S_2_O_8_ (A) ECL curves. (B) Reproducibility of ECL cycles with g-CNQDs/GCE combined with K_2_S_2_O_8_. (C) Cyclic voltammograms.

The ECL signal of g-CNQDs/GCE was then investigated in the presence of the K_2_S_2_O_8_ reactant (Figure 2). Without g-CNQDs or K_2_S_2_O_8_, the ECL signal was weak (Figure 2A). A clear and strong cathodic ECL signal was observed only when g-CNQDs/GCE was combined with K_2_S_2_O_8_. This ECL signal was stable with an intensity that varies only by 1.2% over 12 ECL cycles (Figure 2B). A cyclic voltammetry (CV) analysis of g-CNQDs/GCE confirmed that a reaction responsible for the strong ECL signal occurs between K_2_S_2_O_8_ and g-CNQDs (Figure 2C). In the absence of K_2_S_2_O_8_, no redox peaks were observed indicating that the electrochemical activity of g-CNQDs/GCE was low. In contrast, a bare GCE with K_2_S_2_O_8_ showed a strong peak at −1.25 V (vs SCE) corresponding to K_2_S_2_O_8_ reduction. When g-CNQDs/GCE was combined with K_2_S_2_O_8_, the reduction peak of K_2_S_2_O_8_ decreased indicating that g-CNQDs reacted with K_2_S_2_O_8_ (Dong et al., 2017; Liu et al., 2019).

### ECL-RET biosensor with hairpin DNA probe for EGFR L858R

**Figure 3:**
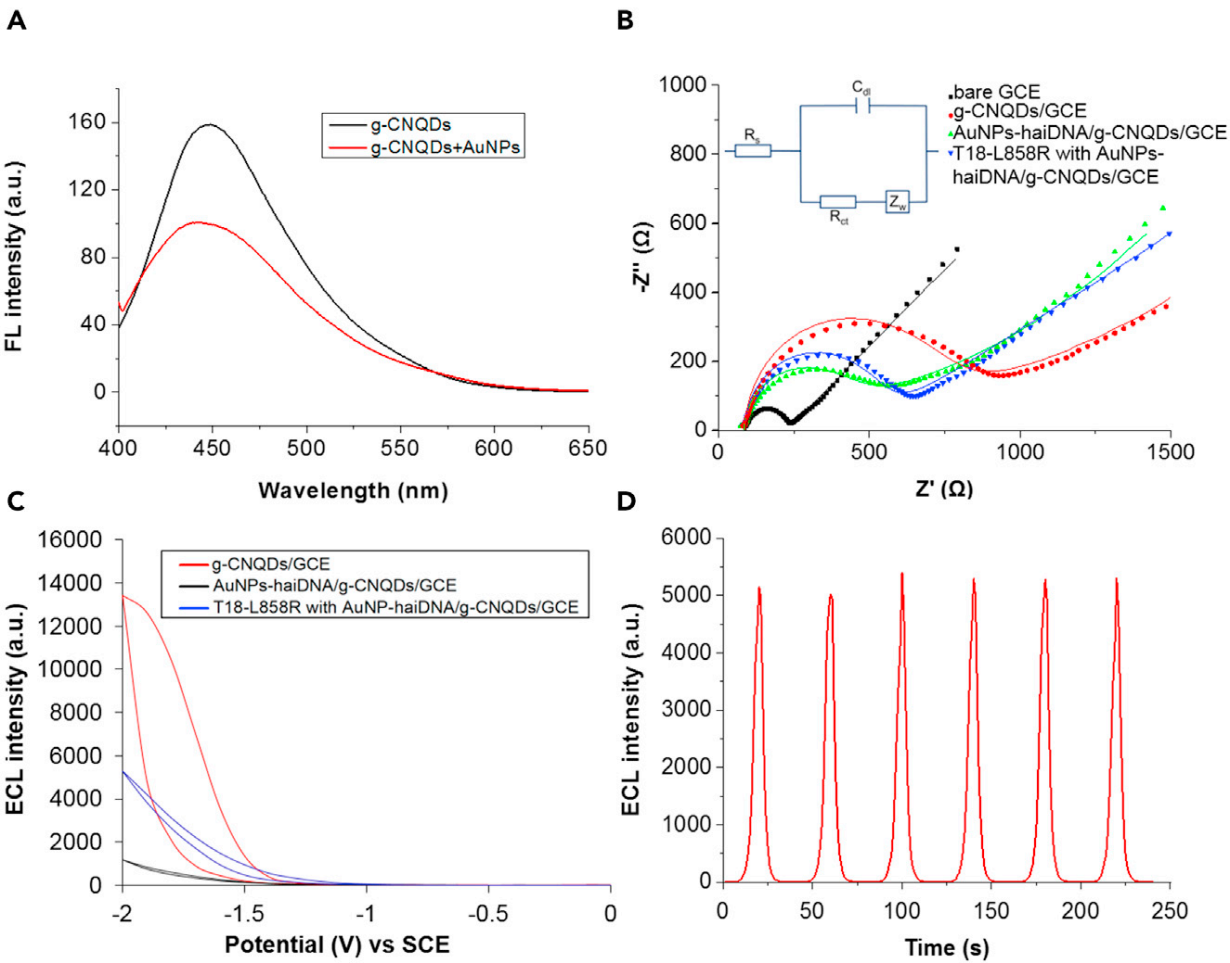
Detection of 18-nucleotide target DNA T18-L858R with the ECL-RET biosensor (A) Fluorescence spectra of g-CNQDs with or without AuNPs. (B and C) Nyquist plots and (C) ECL curves of the ECL-RET biosensor in the presence or not of target DNA T18-L858R. The inset on (B) is the equivalent circuit employed to model the EIS data. R_s_: solution resistance, C_dl:_ double layer capacitance, R_ct_: charge transfer resistance, Z_w_:Warburg diffusion resistance. (D) Reproducibility of ECL cycles with the ECL-RET biosensor in the presence of target DNA T18-L858R.

In the second step of the ECL-RET biosensor fabrication, AuNP-haiDNA probes specific for the detection of DNA molecules coding for the L858R mutation in EGFR associated with NSCLC were attached to the g-CNQDs/GCE (Scheme 1A; Figure S3). In the absence of target DNA, the AuNPs attached to stem-loop haiDNA are in close proximity to g-CNQDs where they smother the ECL signal (Liu et al., 2019). This was demonstrated by fluorescence spectroscopy with a significant decrease of intensity observed upon the addition of AuNPs to g-CNQDs indicating fluorescence resonance energy transfer (FRET) from excited g-CNQDs to AuNPs (Figure 3A). As demonstrated previously, when complementary target DNA is present in the analyte, it anneals with the haiDNA changing its conformation and increasing the distance between the AuNP and the g-CNQDs (Liu et al., 2019; Dong et al., 2017). In turn, this conformational change leads to a stronger ECL signal.

To demonstrate that our system functions correctly to detect the cancer-related mutation, the ECL-RET biosensor was exposed to an 18-nucleotide target ss DNAs bearing the SNP responsible for the EGFR L858R (T18-L858R) and complementary to the loop of the AuNP-haiDNA probe (Figure S4). Electrochemical impedance spectroscopy (EIS) was then employed to assess the impact of T18-L858R DNA and the other main system components on the electrochemical properties of the ECL-RET biosensor (Figure 3B). Nyquist plots drawn from EIS data present semicircles at a high frequency related to the electron transfer limiting process (Tremblay et al., 2020; Han et al., 2018). A Nyquist plot with a smaller semicircle indicates lower charge transfer impedance. The inset on Figure 3B represents the equivalent circuit employed for fitting the EIS data. The different components of this circuit are the solution resistance (R_s_), the charge transfer resistance (R_ct_), the Warburg diffusion resistance (Z_w_), and the double-layer capacitance (C_dl_). As shown in Figure 3B, the experimental EIS data (dot) and the fitted data (line) are consistent. Bare GCE exhibited a small semicircle on the Nyquist plot. When g-CNQDs were coated on GCE, the R_ct_ increased significantly due to the lower conductivity of g-CNQDs. Attaching AuNP-haiDNA to g-CNQDs/GCE lowered the R_ct_ because of the high conductivity of AuNP. In the presence of the T18-L858R target oligonucleotide, the R_ct_ of the ECL-RET biosensor increased, which was caused by the annealing of the target DNA to the AuNP-haiDNA probe resulting in a greater separation of the AuNP from the surface of the g-CNQDs/GCE.

The ECL response of the biosensor was then analyzed at different stages of fabrication and in the presence of target T18-L858R DNA (Figure 3C). The ECL signal emitted by g-CNQDs/GCE with K_2_S_2_O_8_ was significantly reduced when AuNP-haiDNA probes were attached to the surface of the electrode confirming FRET between AuNPs and g-CNQDs. When 100 fM T18-L858R DNA was added to the system, the ECL signal became 4.5 times stronger because of the annealing of T18-L858R DNA to haiDNA resulting in increased distance between AuNPs and g-CNQDs. The ECL signal intensity was stable in the presence of the T18-L858R oligonucleotide with a standard deviation of only 2.5% over six ECL cycles indicating that the ECL-RET biosensor described here is suitable for cancer-related DNA detection (Figure 3D).

### Analytical performance of the biosensor with target DNA T18-L858R

**Figure 4:**
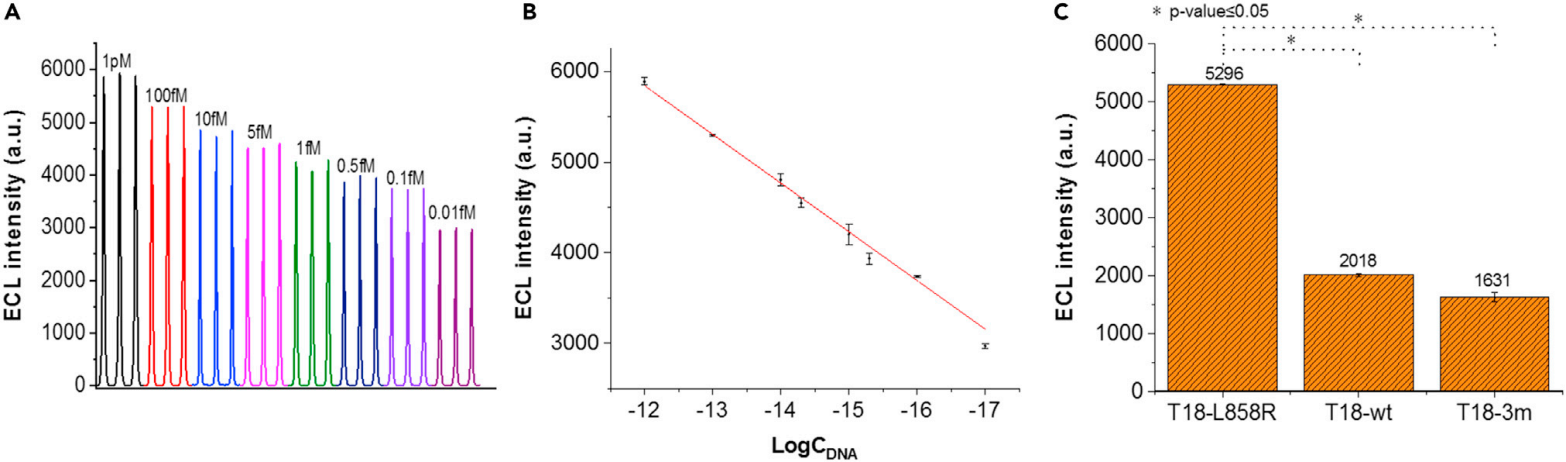
Performance of the ECL-RET biosensor with target DNA T18-L858R (A and B) ECL intensities and (B) logarithmic curves generated with target DNA T18-L858R at concentrations ranging from 0.01 fM to 1 pM. (C) ECL intensities generated by the ECL-RET biosensor in the presence of 100 fM target DNA T18-L858R, wt, or 3m. DNA T18-wt and T18-3m have one and three mismatched nucleotides compared to target DNA T18-L858R, respectively. Data points on (B) and bars on (C) are the mean of at least three replicates with standard deviation. ∗ indicates that the p value is below 0.05.

The linear range and limit of detection of the ECL-RET biosensor were evaluated with different concentrations of the target T18-L858R oligonucleotide diluted in a potassium phosphate buffer at pH 7.4 (Figure 4). The ECL intensity was linearly proportional to the logarithm of target DNA T18-L858R concentrations ranging from 0.01 fM to 1 pM with an R_2_ of 0.9953 (Figure 4B). The limit of detection of the ECL-RET biosensor was 0.0023 fM (3σ). When compared with a similar ECL-RET biosensor developed by Liu et al. (2019) for the detection of ss oligonucleotides unrelated to cancer biology or diagnosis, the system described here was more sensitive (0.0023 fM versus 0.01 fM) and exhibited a wider linear range (0.01 fM to 1 pM compared to 0.02 fM to 0.1 pM). This slightly better performance may be attributed to minor variations in the assembling of the ECL-RET biosensor and/or to the different sequences of the AuNP-haiDNA probes and target DNA molecules involved, which are the major differences between both systems.

The specificity of the ECL-RET biosensor was investigated with 100 fM target DNA molecules harboring either a single-base mismatch corresponding to the wild-type sequence of EGFR gene or three-base mismatches (Figure 4C). The ECL signal was 3.7 and 5.4 times lower in the presence of single-base mismatched or three-base mismatched target DNA, respectively. Thus, the ECL-RET biosensor developed here for the detection of target DNA T18-L858R was specific and was shown to discriminate between target DNA from wild-type EGFR or L858R-mutated EGFRs associated with NSCLC.

### Detection of longer ctDNA-like molecules with ECL-RET biosensor

**Figure 5:**
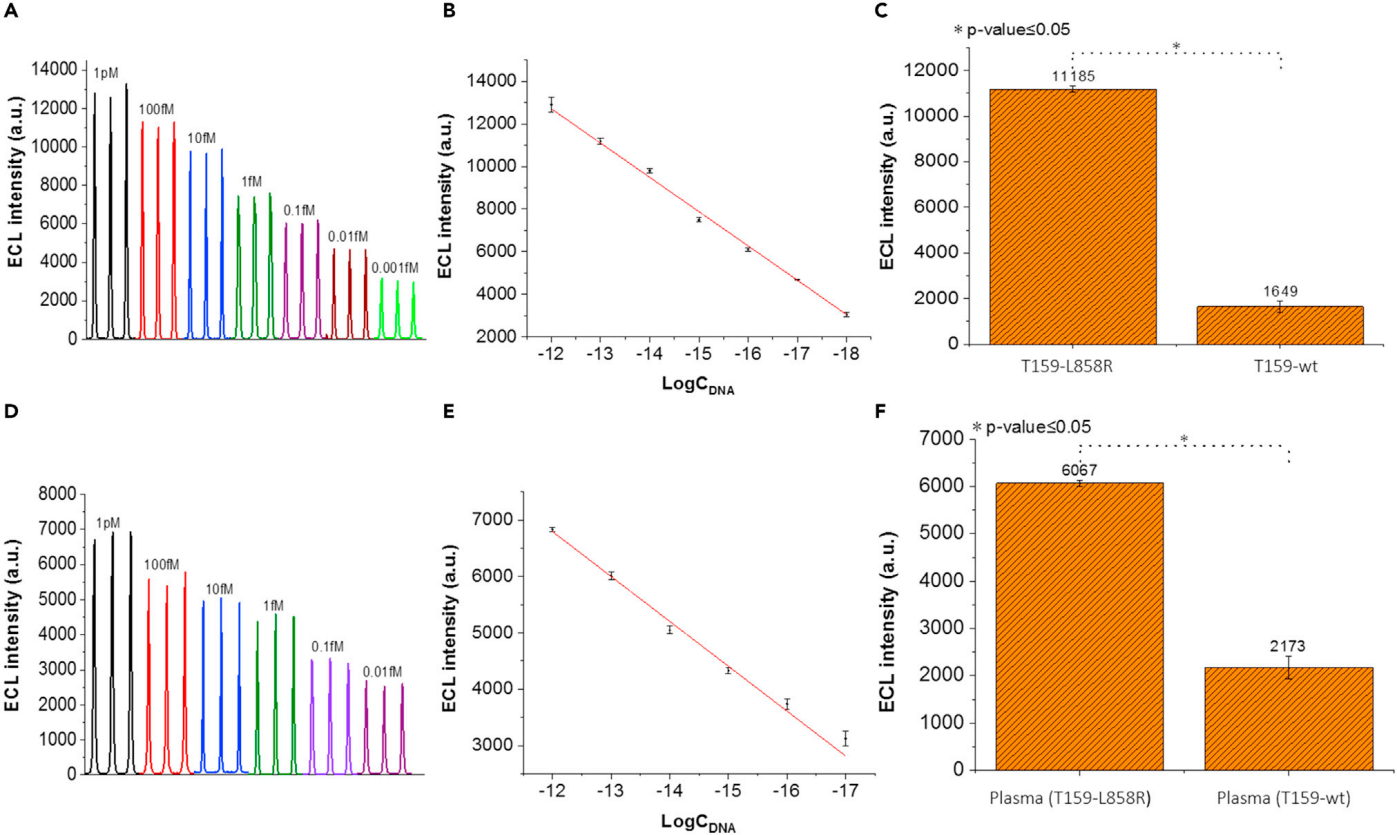
Performance of the ECL-RET biosensor with 159-nucleotide target DNA T159-L858R diluted in potassium phosphate buffer or blood (A and B) ECL intensities and (B) logarithmic curves generated with target DNA T159-L858R diluted in potassium phosphate buffer at concentrations ranging from 0.001 fM to 1 pM. (C) ECL intensities generated by the ECL-RET biosensor in the presence of 100 fM target DNA T159-L858R or wt diluted in potassium phosphate buffer. (D and E) ECL intensities and (E) logarithmic curves generated with target DNA T159-L858R diluted in blood at concentrations ranging from 0.01 fM to 1 pM. (F) ECL intensities generated by the ECL-RET biosensor in the presence of 100 fM target DNA T159-L858R or wt diluted in blood. DNA T159-wt has one mismatched nucleotide compared to target DNA T159-L858R. Data points on (B) and (E) and bars on (C) and (F) are the mean of at least three replicates with standard deviation. ∗ indicates that the p value is below 0.05.

For cancer detection via nucleic acids by liquid biopsy, target ctDNA molecules found in bodily fluids are usually longer than 18 nucleotides. In fact, most ctDNA molecules from plasma are double stranded with a length above 90 bp and up to 320 bp (Mouliere et al., 2018; Rubis et al., 2019). Consequently, the performance of the biosensor developed here was evaluated with a ctDNA-like target ds DNA molecule (T159-L858R) of 159 nucleotides with 79 nucleotides on each side of the SNP responsible for the EGFR L858R mutation (Figures 5; S4). For this purpose, an additional heating step at 95°C for 5 min was added to the biosensing assay prior to the detection step to denature the target ds DNA. ECL intensities were linearly proportional to the logarithm of target DNA T159-L858R diluted in a potassium phosphate buffer (pH 7.4) at concentrations ranging from (Figure 5B) 0.001 fM to 1 pM with an R^2^ of 0.9965. The limit of detection was one order of magnitude lower than with T18-L858R DNA at 0.00055 fM (3σ). The reason for the higher sensitivity of the ECL-RET biosensor for target DNA of 159 nucleotides versus 18 nucleotides is not clear and warrants further investigation.

Results obtained with target DNA T159-L858R and T159-wt indicate that the ECL-RET biosensor was specific (Figure 5C). The ECL signal was 6.8 times lower in the presence of 100 fM T159-wt compared to the same concentration of T159-L858R. The target DNA T159-wt is the wild-type copy of a central section of the human EGFR gene and harbors a single mismatched nucleotide compared to target DNA T159-L858R (Figure S4). In fact, the ECL signal generated by the biosensor in the presence of T159-wt had an intensity comparable to a potassium phosphate buffer control without added target DNA (Figure S5). This indicates that the DNA T159-wt did not trigger the ECL-RET biosensor and that the weak signal observed was caused by unspecific background noise. Overall, these observations show that the ECL-RET biosensor technology developed here is suitable for the detection of target DNA molecules of relevant length for the diagnosis of cancer.

### Detection in blood samples amended with ctDNA-like molecules

The target DNA molecule T159-L858R was diluted into blood samples from a healthy individual to establish if the ECL-RET biosensor can detect and quantify a specific ctDNA-like molecule in a relevant biological fluid (Figure 5). A low-cost and facile pre-treatment was necessary to prepare plasma samples for detection with the ECL-RET biosensor (Breitbach et al., 2014; Umetani et al., 2006). Plasma was isolated from blood by centrifugation and then treated with proteinase K to degrade proteins prior to being heated at 95°C to denature ds DNA. ECL intensities for target DNA T159-L858R in blood plasma were linearly proportional between 0.01 fM to 1 pM with an R^2^ of 0.9911 (Figure 5E). The limit of detection was 0.0023 fM (3 σ). This was ten times less sensitive than what was observed with target DNA T159-L858R in a potassium phosphate buffer, which may be explained by the presence of interfering compounds in the plasma. Nevertheless, these results indicate that the ECL-RET biosensor developed here is functional for the detection and quantification of ctDNA-like molecules in blood plasma over a wide range of concentrations.

When 100 fM target DNA T159-wt was added to the plasma instead of target DNA T159-L858R, the ECL signal was 2.8 times lower demonstrating that the biosensor remains specific when analyzing a biological fluid (Figure 5F). As with phosphate potassium phosphate buffer, the signal observed with T159-wt into plasma was comparable to a plasma control without added target DNA, which indicates that T159-wt DNA did not generate a significant ECL signal (Figure S5). The specificity and wide linear range of the ECL-RET biosensor for the detection of T159-L858R DNA in blood plasma show that this approach could be promising for liquid biopsy and the detection of ctDNA molecules harboring the SNP coding for the L858R mutation associated with NSCLC.

### Conclusions

The ECL-RET biosensor-based assay developed in this proof-of-concept study detected and quantified NSCLC-associated DNA molecules of different lengths diluted into either a potassium phosphate buffer or blood. Interestingly, this technology exhibits several characteristics that could be advantageous for cancer-related liquid biopsy compared to state-of-the-art detection methods including qPCR, ddPCR, and NGS. For instance, both PCR-based and NGS approaches necessitate the isolation of ctDNA from blood plasma with lengthy and expensive DNA purification kits prior to detection and quantification while the ECL-RET biosensor-based assay described here requires only a fast and inexpensive pretreatment with proteinase K (Table S1) (Chen et al., 2017; Sacher et al., 2016; Demuth et al., 2018; Bartels et al., 2017).

Another possible problem with PCR-based approaches, when compared with an ECL-RET biosensor-based assay, is the requirement for a longer DNA region. Primers necessary for PCR amplification must anneal in the region flanking the mutation specific to the ctDNA, which will be recognized by a probe. For example, Oxnard et al. (2014) amplified by ddPCR a DNA fragment in the EGFR gene of 78 nucleotides with 29 and 48 nucleotides on the 5′ and -3′ side, respectively, of the SNP responsible for the L858R mutation (Figure S4) (Oxnard et al., 2014). In comparison, the ECL-RET biosensor can recognize a region of only 18 nucleotides surrounding the L858R-associated SNP. This means that the ECL-RET biosensor could detect shorter ctDNA as well as long ctDNA molecules harboring the EGFR L858R-associated SNP nearer to the 5′ or 3′ ends. With PCR-based approaches, all these target ctDNA molecules would probably not be recognized, which may result in false-negative and/or in the underestimation of the quantity of ctDNA in the plasma.

Clinical diagnostic such as liquid biopsy by NGS also has drawbacks such as the requirement for expensive equipment and highly trained personnel, a complex workflow for data acquisition and analysis, as well as long turnaround times varying between multiple days to weeks (Luthra et al., 2015; Park et al., 2017). In comparison, ctDNA detection by the ECL-RET biosensor-based assay described here has the potential to be less complex with assays completed in several hours.

### Limitations of the study

Critical challenges remain for clinical ctDNA detection by ECL-RET biosensors. The most important one is insufficient sensitivity. Patients at different stages of NSCLC may exhibit between 1 and 50,000 copies per ml of blood of ctDNA carrying the SNP responsible for the EGFR L858R mutation (Sacher et al., 2016; Buder et al., 2019; Zhu et al., 2017) The limit of detection of the ECL-RET biosensor described here was 0.0023 fM or 1,390 copies per ml of blood for EGFR L858R ctDNA-like molecules. This sensitivity would be sufficient for individuals with heavy EGFR L858R ctDNA loads. However, for detection in individuals with lower ctDNA load such as in patients with early-stage cancer, the ECL biosensor will require optimization to augment its sensitivity. Multiple components of the ECL-RET biosensor can be investigated to improve its sensitivity and performance including the length and density of the haiDNA probe, the incubation parameters, as well the type of QDs and plasmonic NPs. Other aspects of DNA detection by ECL-RET biosensors can also be enhanced for liquid biopsies such as the implementation of multiplexing for the simultaneous detection of ctDNA carrying different cancer-related mutations and the shortening of the assay duration. While the ECL-RET biosensing assay necessitates two hours for the annealing of the target DNA to the probe, other electrochemical approaches sensing cell-free nucleic acids performed this step in as little as 15 to 20 min (Das et al., 2015; Chen et al., 2021).

## STAR★Methods

### Key resources table

**Table:** 

REAGENT or RESOURCE	SOURCE	IDENTIFIER
**Biological samples**		

Healthy adult blood	Zhongnan Hospital of Wuhan University	N/A

**Chemicals, peptides, and recombinant proteins**		

Sodium citrate	Nanjing Chemical Reagent Co.	Cat#XW19680422
K_2_S_2_O_8_	Nanjing Chemical Reagent Co.	Cat#10017480
HAuCl_4_·4H_2_O	Shanghai Chemical Reagent Co.	Cat#XW169612541
Thioglycolic acid	Sigma-Aldrich	Cat#T3758
Tris(2-carboxyethyl)phosphine (TCEP)	Thermo Fisher Scientific	Cat#PI20490
(3-aminopropyl)trimethoxysilane	Aladdin Reagent Co	Cat#281778
poly(diallyldimethylammoniumchloride) (PDDA) (20 wt% in water, MW=200,000–350,000)	Aladdin Reagent Co	Cat#409014
Urea	Sinopharm Group Chemical Reagent Co.	Cat#1023228
NaBH_4_	Sinopharm Group Chemical Reagent Co.	Cat#80115865
Proteinase K	Sigma-Aldrich	Cat#P4850
glassy carbon electrode	CH Instruments	Cat#CHI104
Ag/AgCl (3.0 M KCl) reference electrode	CH Instruments	Cat#CHI111
K_2_CO_3_	Sinopharm Group Chemical Reagent Co.	Cat#10016118
Tween 20	Sinopharm Group Chemical Reagent Co.	Cat#30189328
EDTA	Sigma-Aldrich	Cat#E6758
Tris base	Sigma-Aldrich	Cat#T1503

**Oligonucleotides**		

5’-NH_2_-(CH_2_)_6_-GGAAGACAGTTTGGCCCGCCCAA ATCTTCC-(CH_2_)_6_-SH-3’ hairpin DNA probe	Sangon Biotech	N/A
T18-L858R: 5’-TTTGGGCGGGCCAAACTG-3’	Sangon Biotech	N/A
T18-wt (single-base mismatched): 5’-TTTGGGCTGGCCAAACTG-3’	Sangon Biotech	N/A
T18-3m (three-base mismatched): 5’-TTAGGGCTGGCCAATCTG-3’	Sangon Biotech	N/A

**Recombinant DNA**		

ds DNA T159-L858R: 5’-CGCTTGG TGCACCGCGACCTGGCAGC CAGGAACGTACTGGTGAA AACACCGCAGCATGTCAA GATCACAGATTTTGGGCG GGCCAAACTGCTGGGTGC GGAAGAGAAAGAATACCA TGCAGAAGGAGGCAAAG TGCCTATCAAGTGGATGGC ATTGGAA-3’	Sangon Biotech	N/A
ds DNA T159-wt: 5’-CGCT TGGTGCACCGCG ACCTGGCAGCCAGGAAC GTACTGGTGAAAACACC GCAGCATGTCAAGATCAC AGATTTTGGGCTGGCCAA ACTGCTGGGTGCGGAAGA GAAAGAATACCATGCAG AAGGAGGCAAAGTGCC TATCAAGTGGATGGCATTGGAA-3’	Sangon Biotech	N/A

**Software and algorithms**		

ZView	AMETEK Scientific Instruments	N/A

**Other**		

Ultra-weak luminescence analyzer	Institute of Biophysics of the Chinese Academy of Science	Cat#BPCL-GP21Q
Electrochemical workstation	CH Instruments	Cat#CHI660E

### Resource availability

#### Lead contact

Further information and requests for resources and reagents should be directed to and will be fulfilled by the lead contact, Tian Zhang (tzhang@whut.edu.cn).

#### Materials availability

This study did not generate new unique reagents.

### Experimental model and subject details

The study was approved by the Medical Ethics Committee of Zhongnan Hospital of Wuhan University. Blood was sampled from a healthy 25-year old male volunteer.

### Method details

#### Characterization of the ECL-RET biosensor

ECL signals were quantified with a BPCL-GP21Q ultra-weak luminescence analyzer purchased from the Institute of Biophysics of the Chinese Academy of Science (Beijing, China). The ECL experiments were carried out with a three-electrode system comprising a GCE with a diameter of 3 mm as the support for the working electrode, a Pt plate as the counter-electrode, and an Ag/AgCl (3.0 M KCl) reference electrode. UV-Vis absorption spectra were acquired with a UV-3600 UV-vis-NIR spectrophotometer (Shimadzu, Kyoto, Japan). Fluorescence spectra were obtained with a F-7000 fluorescence spectrophotometer (Hitachi, Tokyo, Japan). The Fourier transform infrared spectroscopy (FTIR) spectrum of g-CNQDs was recorded with a Nicolet iS5 FTIR spectrometer (Thermo Fisher Scientific, Waltham, MA, USA) in the 450 to 4000 cm^−1^ range. The X-ray diffraction (XRD) spectrum of g-CNQDs was obtained with a D8 ADVANCE powder X-ray diffractometer (Bruker, MA, USA) in the range of 10–80° with Cu Kα radiation (2θ). Transmission electron microscopy (TEM) images were taken with a JEM-2100F (JEOL, Akishima, Japan) field emission electron microscope at an accelerating voltage of 200 kV.

#### Hairpin DNA probe and target DNA molecules

The haiDNA probe, the ss 18-nucleotide target DNA molecules, and the ds 159-nucleotide target DNA molecules were synthesized by Sangon Biotech (Shanghai, China).

#### Preparation of g-CNQDs

g-CNQDs were synthesized by the low-temperature solid-phase technique as previously described with several modifications (Zhou et al., 2013). Briefly, 1.68 mmol urea and 0.28 mmol sodium citrate were mixed in a 6:1 molar ratio and ground in an agate mortar. The mixture was then heated at 180°C in a stainless-steel autoclave for one hour. After cooling down to room temperature, the product, which exhibits a light-yellow color, was dispersed into anhydrous ethanol prior to being centrifuged at 17,000 × g for 10 min. Ethanol washing was repeated five times. In the final step, the product was further purified via dialysis in ultrapure water for 24 hours.

#### AuNPs synthesis and attachment to hairpin DNA probe

AuNPs were prepared as described previously (Ding et al., 2006). In brief, 3 ml of 1% (w/v) HAuCl_4_ was diluted into 200 ml ultrapure water and stirred vigorously at 4°C. During stirring, 1 ml of 0.2 M K_2_CO_3_ was added. Subsequently, 9 ml NaBH_4_ (0.5 mg ml^−1^) was mixed into the solution, which was stirred for an additional 5 min. The resulting red-colored solution was stored at 4°C until usage.

For attachment to AuNPs, haiDNA molecules were first activated by mixing 10 μl of 1 μM haiDNA with 0.15 μl of 10 mM TCEP solubilized in 10 mM potassium phosphate buffer with 100 mM NaCl at pH 7.4 followed by incubation for one hour at room temperature as described previously (Dong et al., 2017). Subsequently, 100 μl AuNPs was added to the mixture followed by incubation at 25°C for 30 min. In the last step, 10 μl of 10 mM thioglycolic acid was added and the solution was incubated for 2 hours at 25°C to limit unspecific retention of AuNPs. The mixture was kept at 4°C until further usage.

#### Blood plasma preparation

Blood samples were centrifuged at 1,500 × g for 10 min at 4°C to separate plasma, which was stored at −80°C until further usage. For ctDNA-like molecules measurement, T159-L858R or T159-wt DNA was diluted at different concentrations in blood samples prior to plasma separation. For sensing with the ECL-RET biosensor, plasma samples were pre-treated to degrade proteins and to denature ds DNA by employing a method described previously but with several modifications (Breitbach et al., 2014; Umetani et al., 2006). Briefly, 20 μl plasma was mixed with 5 μl concentrated proteinase K buffer made of 125 mM Tris-Cl pH 8.0, 6.25% (v/v) Tween 20, and 2.5 mM EDTA. 10 μg proteinase K was then added to the mixture, which was incubated at 50°C for 20 minutes. In the last step, the solution was incubated at 95°C for 5 minutes to simultaneously inactivate proteinase K and denature ds DNA.

#### Fabrication of ECL-RET biosensor and DNA detection

In the first step of the working electrode fabrication for the ECL-RET biosensor, the GCE was polished with alumina paste of 0.3 and 0.05 μm prior to being washed with 50% ethanol, nitric acid, and ultrapure water (Liu et al., 2019). After drying, 10 μl of dialyzed g-CNQDs was deposited on the GCE followed by incubation at 37°C for one hour. Subsequently, 10 μl PDDA was added drop-wise on the g-CNQDs/GCE, which was then incubated in a desiccator at room temperature for one hour. In the next step, 10 μl AuNP-haiDNA was deposited on the g-CNQDs/GCE followed by a one-hour incubation at 37°C. The as-prepared working electrode was then washed with 10 mM potassium phosphate buffer (pH 7.4) to remove unbound AuNP-haiDNA.

For detection, 10 μl target DNA samples were deposited on the working electrode of the ECL-RET biosensor followed by a two-hour incubation at 37°C within an electrolyte solution made of 100 mM potassium phosphate buffer (pH 7.4) with 100 mM K_2_S_2_O_8_. ECL signals were then measured with an ultra-weak luminescence analyzer.

#### Cyclic voltammetry and electrochemical impedance spectroscopy

The three-electrode system described beforehand was employed for cyclic voltammetry (CV) and electrochemical impedance spectroscopy (EIS). CV was completed in a potential window of 0 to −2000 mV versus Ag/AgCl with a scan rate of 0.1 V s^−1^. 15 ml of 100 mM potassium phosphate buffer (pH 7.4) was used as the electrolyte with or without 100 mM K_2_S_2_O_8_. EIS spectra were obtained within a scan range of 100 kHz to 0.01 Hz at a 5 mV amplitude. For EIS, the electrolyte solution comprised 10 mM K_3_[Fe(CN)_6_]/K_4_[Fe(CN)_6_] (1:1) solubilized in a 100 mM potassium phosphate buffer (pH 7.4) containing 0.1 M KCl. The ZView software was employed to fit the Nyquist plots resulting from EIS experiments with the equivalent circuit.

### Quantification and statistical analysis

#### Limit of detection calculation

The limit of detection of the ECL-RET biosensor was measured with the commonly-used 3σ method (Yang et al., 2020; Fan et al., 2020; Liang et al., 2016; Cai et al., 2021). Briefly, for each condition, the standard deviation of samples with the lowest concentration is calculated and multiply by three to obtain the 3σ limit of detection.

#### Statistical significance

In this study, results are considered to be statistically significant when the p value calculated with the Student's t-test is below or equal to 0.05.

## Data Availability

• All data are reported in the main text or in the supplemental information of this work. • This study does not report original code. • Any additional information required to reanalyze the data reported in this paper is available from the lead contact upon request.
