# Supplementary material for: An electrochemiluminescence resonance energy transfer biosensor for the detection of circulating tumor DNA from blood plasma
Source: iScience. 2021 Aug 21;24(9):103019. doi: 10.1016/j.isci.2021.103019 (PMC8426273; doi:10.1016/j.isci.2021.103019)
Supplement: Document S1. Figures S1–S5 and Tables S1 and S2 [file mmc1.pdf]

iScience, Volume *24*

Supplemental information

An electrochemiluminescence resonance energy transfer biosensor for the detection of circulating tumor DNA from blood plasma

Xidong Yang, Meiyan Liao, Hanfei Zhang, JinBo Gong, Fan Yang, Mengying Xu, Pier-Luc Tremblay, and Tian Zhang

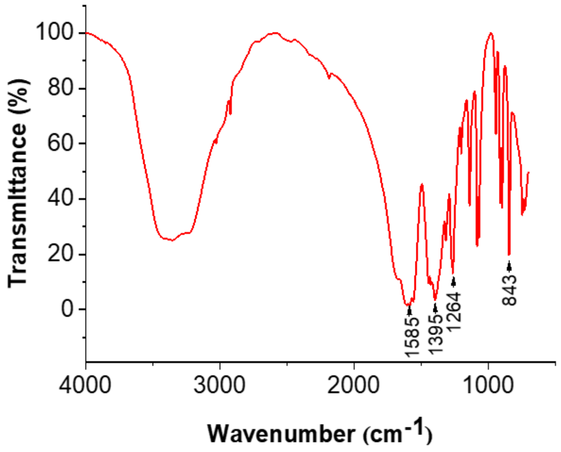

Figure S1. FTIR spectrum of g-CNQDs. Related to Figure 1. The band detected at ca. 843 cm^{-1} corresponds to heptazine units. The two bands at 1264 cm^{-1} and 1395 cm^{-1} are attributed to aromatic C-N vibrations. The intense peak at 1585 cm^{-1} characteristic of asymmetric C=O stretching indicates that g-CNQDs contain carboxylate anions. The broad peaks between 3100 and 3500 cm^{-1} are distinctive of N-H and O-H stretching (Liu et al., 2011; Zhang et al., 2019).

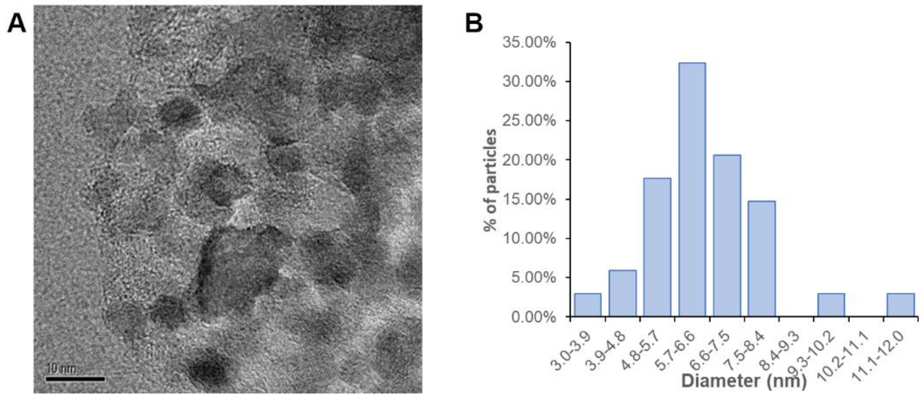

Figure S2. Particle size distribution of g-CNQDs. Related to Figure 1. (A) TEM image and (B) particle size distribution.

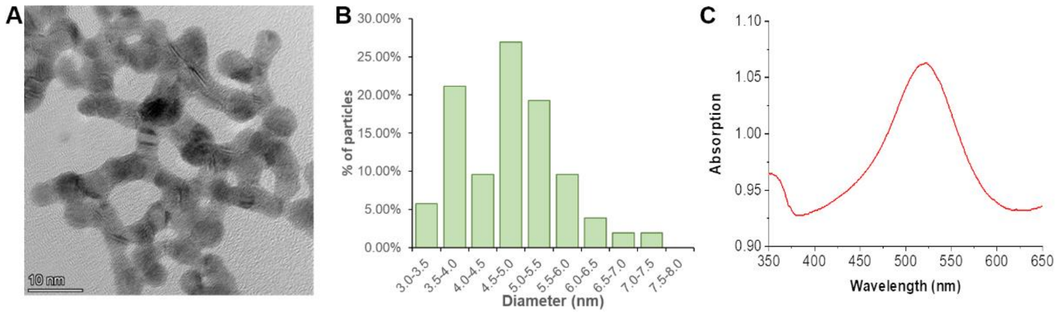

Figure S3. The characterization of AuNPs. Related to Scheme 1. (A) TEM image, (B) particle size distribution, and (C) UV–vis absorption spectrum of AuNPs. The TEM image shows AuNPs with good dispersity and uniformity as well as a diameter ranging from 3.2 nm to 7.0 nm with an average particle diameter of 4.7 nm (n=30). The UV-Vis absorption spectrum exhibits a peak at 521nm distinctive of AuNPs as reported previously (He et al., 2005).

ATGCGACCCTCCGGGACGGCCGGGGCAGCGCTCCTGGCGCTGCTGGCTGCGCTCTGCCCGGCGAGTCGGGCTCTGGAGGAAAAGA
AAGTTTGCCAAGGCACGAGTAACAAGCTCACGCAGTTGGGCACTTTTGAAGATCATTTTCTCAGCCTCCAGAGGATGTTCAATAA
CTGTGAGGTGGTCCTTGGGAATTTGGAAATTACCTATGTGCAGAGGAATTATGATCTTTCCTTCTTAAAGACCATCCAGGAGGTG
GCTGGTTATGTCCTCATTGCCCTCAACACAGTGGAGCGAATTCCTTTGGAAAACCTGCAGATCATCAGAGGAAATATGTACTACG
AAAATTCCTATGCCTTAGCAGTCTTATCTAACTATGATGCAAATAAAACCGGACTGAAGGAGCTGCCCATGAGAAATTTACAGGA
AATCCTGCATGGCGCCGTGCGGTTCAGCAACAACCCTGCCCTGTGCAACGTGGAGAGCATCCAGTGGCGGGACATAGTCAGCAGT
GACTTTCTCAGCAACATGTCGATGGACTTCCAGAACCACCTGGGCAGCTGCCAAAAGTGTGATCCAAGCTGTCCCAATGGGAGCT
GCTGGGGTGCAGGAGAGGAGAACTGCCAGAAACTGACCAAAATCATCTGTGCCCAGCAGTGCTCCGGGCGCTGCCGTGGCAAGTC
CCCCAGTGACTGCTGCCACAACCAGTGTGCTGCAGGCTGCACAGGCCCCCGGGAGAGCGACTGCCTGGTCTGCCGCAAATTCCGA
GACGAAGCCACGTGCAAGGACACCTGCCCCCCACTCATGCTCTACAACCCCACCACGTACCAGATGGATGTGAACCCCGAGGGCA
AATACAGCTTTGGTGCCACCTGCGTGAAGAAGTGTCCCCGTAATTATGTGGTGACAGATCACGGCTCGTGCGTCCGAGCCTGTGG
GGCCGACAGCTATGAGATGGAGGAAGACGGCGTCCGCAAGTGTAAGAAGTGCGAAGGGCCTTGCCGCAAAGTGTGTAACGGAATA
GGTATTGGTGAATTTAAAGACTCACTCTCCATAAATGCTACGAATATTAAACACTTCAAAAACTGCACCTCCATCAGTGGCGATC
TCCACATCCTGCCGGTGGCATTTAGGGGTGACTCCTTCACACATACTCCTCCTCTGGATCCACAGGAACTGGATATTCTGAAAAC
CGTAAAGGAAATCACAGGGTTTTTGCTGATTCAGGCTTGGCCTGAAAACAGGACGGACCTCCATGCCTTTGAGAACCTAGAAATC
ATACGCGGCAGGACCAAGCAACATGGTCAGTTTTCTCTTGCAGTCGTCAGCCTGAACATAACATCCTTGGGATTACGCTCCCTCA
AGGAGATAAGTGATGGAGATGTGATAATTTCAGGAAACAAAAATTTGTGCTATGCAAATACAATAAACTGGAAAAAACTGTTTGG
GACCTCCGGTCAGAAAACCAAAATTATAAGCAACAGAGGTGAAAACAGCTGCAAGGCCACAGGCCAGGTCTGCCATGCCTTGTGC
TCCCCCGAGGGCTGCTGGGGCCCGGAGCCCAGGGACTGCGTCTCTTGCCGGAATGTCAGCCGAGGCAGGGAATGCGTGGACAAGT
GCAACCTTCTGGAGGGTGAGCCAAGGGAGTTTGTGGAGAACTCTGAGTGCATACAGTGCCACCCAGAGTGCCTGCCTCAGGCCAT
GAACATCACCTGCACAGGACGGGGACCAGACAACTGTATCCAGTGTGCCCACTACATTGACGGCCCCCACTGCGTCAAGACCTGC
CCGGCAGGAGTCATGGGAGAAAACAACACCCTGGTCTGGAAGTACGCAGACGCCGGCCATGTGTGCCACCTGTGCCATCCAAACT
GCACCTACGGATGCACTGGGCCAGGTCTTGAAGGCTGTCCAACGAATGGGCCTAAGATCCCGTCCATCGCCACTGGGATGGTGGG
GGCCCTCCTCTTGCTGCTGGTGGTGGCCCTGGGGATCGGCCTCTTCATGCGAAGGCGCCACATCGTTCGGAAGCGCACGCTGCGG
AGGCTGCTGCAGGAGAGGGAGCTTGTGGAGCCTCTTACACCCAGTGGAGAAGCTCCCAACCAAGCTCTCTTGAGGATCTTGAAGG
AAACTGAATTCAAAAAGATCAAAGTGCTGGGCTCCGGTGCGTTCGGCACGGTGTATAAGGGACTCTGGATCCCAGAAGGTGAGAA
AGTTAAAATTCCCGTCGCTATCAAGGAATTAAGAGAAGCAACATCTCCGAAAGCCAACAAGGAAATCCTCGATGAAGCCTACGTG
ATGGCCAGCGTGGACAACCCCCACGTGTGCCGCCTGCTGGGCATCTGCCTCACCTCCACCGTGCAGCTCATCACGCAGCTCATGC
CCTTCGGCTGCCTCCTGGACTATGTCCGGGAACACAAAGACAATATTGGCTCCCAGTACCTGCTCAACTGGTGTGTGCAGATCGC
AAAGGGCATGAACTACTTGGAGGACCGTCGCTTGGTGCACCGCGACCTGGCAGCCAGGAACGTACTGGTGAAAACACCGCAGCAT
GTCAAGATCACAGAT*TTTGGGC**T**GGCCAAACTG*CTGGGTGCGGAAGAGAAAGAATACCATGCAGAAGGAGGCAAAGTGCCTATC
AAGTGGATGGCATTGGAATCAATTTTACACAGAATCTATACCCACCAGAGTGATGTCTGGAGCTACGGGGTGACTGTTTGGGAGT
TGATGACCTTTGGATCCAAGCCATATGACGGAATCCCTGCCAGCGAGATCTCCTCCATCCTGGAGAAAGGAGAACGCCTCCCTCA
GCCACCCATATGTACCATCGATGTCTACATGATCATGGTCAAGTGCTGGATGATAGACGCAGATAGTCGCCCAAAGTTCCGTGAG
TTGATCATCGAATTCTCCAAAATGGCCCGAGACCCCCAGCGCTACCTTGTCATTCAGGGGGATGAAAGAATGCATTTGCCAAGTC
CTACAGACTCCAACTTCTACCGTGCCCTGATGGATGAAGAAGACATGGACGACGTGGTGGATGCCGACGAGTACCTCATCCCACA
GCAGGGCTTCTTCAGCAGCCCCTCCACGTCACGGACTCCCCTCCTGAGCTCTCTGAGTGCAACCAGCAACAATTCCACCGTGGCT
TGCATTGATAGAAATGGGCTGCAAAGCTGTCCCATCAAGGAAGACAGCTTCTTGCAGCGATACAGCTCAGACCCCACAGGCGCCT
TGACTGAGGACAGCATAGACGACACCTTCCTCCCAGTGCCTGAATACATAAACCAGTCCGTTCCCAAAAGGCCCGCTGGCTCTGT
GCAGAATCCTGTCTATCACAATCAGCCTCTGAACCCCGCGCCCAGCAGAGACCCACACTACCAGGACCCCCACAGCACTGCAGTG
GGCAACCCCGAGTATCTCAACACTGTCCAGCCCACCTGTGTCAACAGCACATTCGACAGCCCTGCCCACTGGGCCCAGAAAGGCA
GCCACCAAATTAGCCTGGACAACCCTGACTACCAGCAGGACTTCTTTCCCAAGGAAGCCAAGCCAAATGGCATCTTTAAGGGCTC
CACAGCTGAAAATGCAGAATACCTAAGGGTCGCGCCACAAAGCAGTGAATTTATTGGAGCATGA

EGFR wt **EGFR L858R**

C**T**G ⟶ C**G**G

Figure S4. Coding DNA sequence of the Human Epidermal Growth Factor Receptor (EGFR). Related to Figure 3, Figure 4, and Figure 5. The single nucleotide polymorphism (T→G) responsible for the EGFR L858R mutation is in bold red. The codon CTG corresponding to leucine (L) changes to the codon CGG corresponding to arginine (R) in EGFR L858R is in red. The sequence corresponding to the target DNA T18 is italicized. The sequence corresponding to the target DNA

T159 is underlined. The DNA sequences corresponding to the primers designed by Oxnard et al., (2014) for ddPCR are in purple. The DNA sequence corresponding to the probe designed by Oxnard et al. (2014) for ddPCR is highlighted in gray.

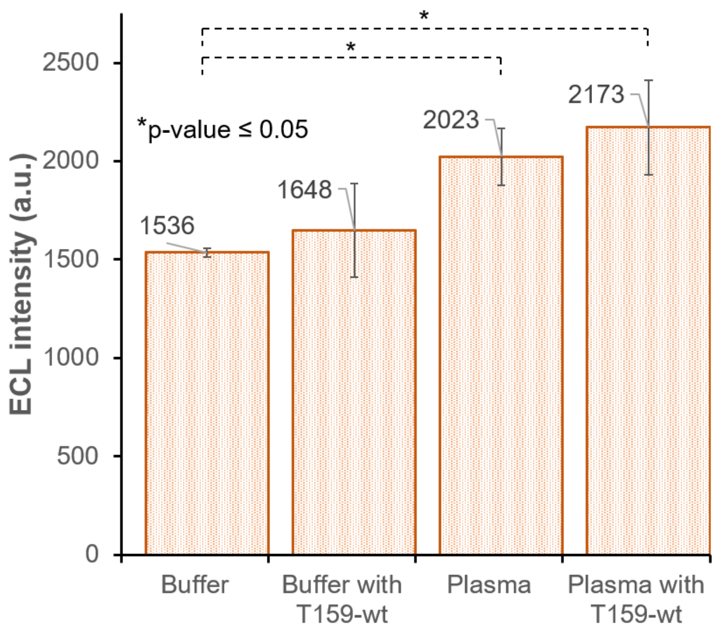

Figure S5. ECL intensities generated by the ECL-RET biosensor with potassium phosphate buffer and plasma without added target DNA or with 100 fM T159-wt. Related to Figure 5. These results demonstrate that DNA T159-wt is not detected by the ECL-RET biosensor. Additionally, the buffer- and plasma-only controls show that the background signal is stronger with plasma than with potassium phosphate buffer, which is possibly related to chemicals in the plasma impacting the ECL signal of the sensor system. Bars are the mean of at least three replicates with standard deviation. * indicates that the p-value is below 0.05.

**Table S1. Examples of widely-used standard ctDNA preparation and detection methods. Related to Scheme 1.**			
**Mutation/Cancer**	**Detection method**	**Sample preparation[a]**	**Reference**
Multiple mutations/triple-negative breast cancer	Ion proton NGS[b]	Plasma DNA isolated with QIAamp Circulating Nucleic Acid Kit	(Chen et al., 2017)
EGFR and KRAS mutations/NSCLC[c]	ddPCR[d]	Plasma DNA isolated with QIAmp circulating nucleic acid kit	(Sacher et al., 2016)
BRAF, KRAS and NRAS/colorectal cancer	qPCR[e]/NGS	QIAamp DNA Mini Kit/ Somatic 1 Master Kit/ QIAamp DSP Virus Spin Kit/ NucleoSpin Plasma XS Kit /Agencourt Genfind v2 Kit	(Beránek et al., 2016)
KRAS mutations/metastatic colorectal cancer	ddPCR/NGS	Therascreen DXS KRASmutation kit/ QIAamp Circulating Nucleic Acid Kit / Oncomine Solid Tumor DNA kit	(Demuth et al., 2018)
EGFR mutations/NSCLC	ddPCR/NGS/ qPCR	QIAamp circulating Nucleic Acid Kit / Qubit 2.0 Fluorometer with Qubit dsDNA HS (High Sensitivity) Assay Kit	(Bartels et al., 2017)

[a]No expensive kit is required to extract circulating nucleic acids with the ECL-RET sensing assay described in this work.
[b]NGS: Next-generation sequencing.
[c]NSCLC: Non-small cell lung cancer.
[d]ddPCR: Droplet digital PCR.
[e]qPCR: Quantitative PCR.

Table S2. Examples of electrochemical or electrochemiluminescent sensing assays developed for the detection of ctDNA in blood plasma or sera. Related to Scheme 1.				
**Target DNA**	**Electrochemical sensor**	**Sample preparation**	**Sensitivity/ Time**	**Reference**
ctDNA from lung cancer patient with mutated KRAS or melanoma cancer patients with mutated BRAF	Amplification-free DNA clutch probes with peptide nucleic acid (PNA) clamps and probes	-DNA purified from sera with a Norgen plasma/serum circulating DNA purification kit<br>-RNase A treatment<br>-DNA heated at 90 °C for 2 min	-Mutations detected at 0.01% relative to wild type<br>-Assay time: Ca. 50 min.	(Das et al., 2016)
PIK3CA E545K ctDNA from blood and pleural effusion samples of cancer patients	Nest hybridization chain reaction (HCR) with dumbbell-shaped DNA probes	-Cell free DNA in biological samples isolated with the QIAamp Circulating Nucleic Acid kit	-Linear range: 5 pM to 0.5 nM<br>-Detection limit of 3 pM<br>-Assay time: Ca. two hours	(Huang et al., 2020)
PIK3CA E545K ctDNA from blood samples of breast cancer patients	Wheel-like catalytic hairpin assembly with frame hybridization chain reaction	-Cell free DNA in biological samples isolated with the QIAamp Circulating Nucleic Acid kit	-Linear range: 10 fM to 5 nM<br>-Detection limit of 8.3 fM<br>-Assay time: Ca. three hours	(Luo et al., 2021)
101-nucleotide synthetic ctDNA associated with NSCLC spiked in 50% blood	Methylene-blue-DNA probe modified gold-coated magnetic Nanoparticles	-Not specified	-Linear range[a]: 200 aM to 20 nM (with ctDNA in PBS<br>-Detection limit[a]: 5 fM (with ctDNA in PBS)<br>-Assay time: 20 min.	(Chen et al., 2021)
159-nucleotide synthetic EGFR L858R ctDNA associated with	Amplification-free g-CNQDs EC-RET system	-After blood centrifugation, plasma was	-Linear range: 0.01 fM to 1 pM	This work

NSCLC spiked in blood		treated with proteinase K<br>-For DNA denaturation, plasma was heated at 95 ºC for 5 minutes	-Detection limit: 0.0023 fM	
[a]Linear range and detection limit are not indicated with synthetic ctDNA spiked in 50% blood.				

References

Bartels, S., Persing, S., Hasemeier, B., Schipper, E., Kreipe, H., and Lehmann, U. (2017). Molecular analysis of circulating cell-free DNA from lung cancer patients in routine laboratory practice: A cross-platform comparison of three different molecular methods for mutation detection. J Mol Diagn *19*, 722–732.

Beránek, M., Sirák, I., Vošmik, M., Petera, J., Drastíková, M., and Palička, V. (2016). Carrier molecules and extraction of circulating tumor DNA for next generation sequencing in colorectal cancer. Acta Medica (Hradec Kralove) *59*, 54–58.

Chen, D., Wu, Y., Hoque, S., Tilley, R.D., and Gooding, J.J. (2021). Rapid and ultrasensitive electrochemical detection of circulating tumor DNA by hybridization on the network of gold-coated magnetic nanoparticles. Chem. Sci. *12*, 5196–5201.

Chen, Y.-H., Hancock, B.A., Solzak, J.P., Brinza, D., Scafe, C., Miller, K.D., and Radovich, M. (2017). Next-generation sequencing of circulating tumor DNA to predict recurrence in triple-negative breast cancer patients with residual disease after neoadjuvant chemotherapy. NPJ Breast Cancer *3*, 24.

Das, J., Ivanov, I., Sargent, E.H., and Kelley, S.O. (2016) DNA clutch probes for

circulating tumor DNA analysis. J. Am. Chem. Soc. *138*, 11009–11016.

Demuth, C., Spindler, K.-L.G., Johansen, J.S., Pallisgaard, N., Nielsen, D., Hogdall, E., Vittrup, B., and Sorensen, B.S. (2018). Measuring KRAS mutations in circulating tumor DNA by droplet digital PCR and next-generation sequencing. Transl. Oncol. *11*, 1220–1224.

He, Y.Q., Liu, S.P., Kong, L., and Liu, Z.F. (2005). A study on the sizes and concentrations of gold nanoparticles by spectra of absorption, resonance Rayleigh scattering and resonance non-linear scattering. Spectrochim. Acta A Mol. Biomol. Spectrosc. *61*, 2861–2866.

Huang, Y., Tao, M., Luo, S., Zhang, Y., Situ, B., Ye, X., Chen, P., Jiang, X., Wang, Q., and Zheng, L. (2020). A novel nest hybridization chain reaction based electrochemical assay for sensitive detection of circulating tumor DNA. Anal. Chim. Acta *1107*, 40–47.

Liu, J., Zhang, T., Wang, Z., Dawson, G., and Chen, W. (2011). Simple pyrolysis of urea into graphitic carbon nitride with recyclable adsorption and photocatalytic activity. J. Mater. Chem. *21*, 14398–14401.

Luo, S., Zhang, Y., Huang, G., Situ, B., Ye, X., Tao, M., Huang, Y., Li, B., Jiang, X., Wang, Q., and Zheng, L. (2021). An enzyme-free amplification strategy for sensitive assay of circulating tumor DNA based on wheel-like catalytic hairpin assembly and frame hybridization chain reaction. Sens. and Actuators B Chem. *338*, 129857.

Oxnard, G.R., Paweletz, C.P., Kuang, Y., Mach, S.L., O'Connell, A., Messineo, M.M.,

Luke, J.J., Butaney, M., Kirschmeier, P., Jackman, D.M., and Jänne, P.A. (2014). Noninvasive detection of response and resistance in EGFR-mutant lung cancer using quantitative next-generation genotyping of cell-free plasma DNA. Clin. Cancer Res. *20*, 1698–1705.

Sacher, A.G., Paweletz, C., Dahlberg, S.E., Alden, R.S., O'Connell, A., Feeney, N., Mach, S.L., Jänne, P.A., and Oxnard, G.R. (2016). Prospective validation of rapid plasma genotyping for the detection of EGFR and KRAS mutations in advanced lung cancer. JAMA Oncol. *2*, 1014–1022.

Zhang, Y., Meng, S., Ding, J., Peng, Q., and Yu, Y. (2019). Transition metal-coordinated graphitic carbon nitride dots as a sensitive and facile fluorescent probe for β-amyloid peptide detection. Analyst *144*, 504–511.
